# Effects of Melatonin on H_2_O_2_-Induced Oxidative Damage of the Granulosa Cells in Hen Ovarian Follicles

**DOI:** 10.3390/genes16040362

**Published:** 2025-03-22

**Authors:** Sheng Wang, Yu Ou, Shengxiao Cao, Xue Sun, Ning Qin, Simushi Liswaniso, Rifu Xu

**Affiliations:** 1 Department of Animal Genetics, Breeding and Reproduction, College of Animal Science and Technology, Jilin Agricultural University, Changchun 130118, China; haojunzai@163.com (S.W.); ouyu12380@mails.jlau.edu.cn (Y.O.); jlaucsxiao@mails.jlau.edu.cn (S.C.); xuesun@jlau.edu.cn (X.S.); ningqin@jlau.edu.cn (N.Q.); smliswaniso@gmail.com (S.L.); 2 Joint Laboratory of Modern Agricultural Technology International Cooperation, Ministry of Education, Jilin Agricultural University, Changchun 130118, China

**Keywords:** chicken, melatonin, granulosa cells, oxidative damage, mitochondrial autophagy

## Abstract

Background: The egg-laying performance of hens is primarily regulated by ovarian follicle growth and development; these follicles are susceptible to oxidative damage caused by excessive reactive oxygen species (ROS). Oxidative damage can lead to follicular atresia and impaired reproductive performance. Melatonin (MT), a known endogenous antioxidant, plays a role in regulating oxidative damage, but its precise mechanisms in mitigating H_2_O_2_-induced oxidative damage via mitophagy regulation in granulosa cells remain unclear. Methods: An in vitro oxidative damage model was established by determining the optimal H_2_O_2_ concentration using CCK-8 fluorescence quantification. The optimal MT concentration was identified through fluorescence quantification and catalase (CAT) activity assays. The protective effects of MT against H_2_O_2_-induced oxidative damage in follicular granulosa cells were investigated using flow cytometry, Western blotting, ELISA, and quantitative fluorescence analysis. Results: An in vitro oxidative damage model was established using H_2_O_2_-induced granulosa cells, characterized by *P53* and *LC3-II* upregulation and *LC3-I* and *BCL-2* downregulation. The optimal MT concentration for reducing cellular injury was determined. MT co-treatment enhanced CAT, GSH, and SOD activities, decreased LC3-II/LC3-I conversion, and increased P62 expression. Furthermore, MT reduced autophagic vesicle formation and restored mitochondrial membrane potential, demonstrating its protective effect against H_2_O_2_-induced oxidative damage. Conclusions: Melatonin alleviates H_2_O_2_-induced oxidative damage in chicken follicular granulosa cells by modulating antioxidant defense, autophagy, and mitochondrial function. These findings provide newer insights to our understanding of the regulatory mechanisms underlying the alleviation of the H_2_O_2_-induced oxidative damage in granulosa cells during ovarian follicle development in chickens.

## 1. Introduction

Follicular development is vital for poultry reproduction, with granulosa cells (GCs) playing a key role in follicular growth, hormone synthesis, and oxidative damage regulation [1,2,3]. The current research suggests that autophagy in GCs may be an important process in follicular atresia induced by oxidative damage [4]. Excessive ROS presence disrupts the balance between oxidative damage and antioxidant defense, causing GCs apoptosis and follicular dysfunction [5,6]. Studies suggest that oxidative damage may affect granulosa cell survival by triggering mitochondrial dysfunction, apoptosis, and autophagy dysregulation [7]. Key regulators like *P53*, *LC3*, and *BCL-2* are involved in oxidative damage-induced apoptosis and autophagy, impacting ovarian function [8]. Hydrogen peroxide is commonly employed for establishing in vitro oxidative damage models, owing to its capacity to generate controlled cellular injury [9]. Experimental evidence demonstrates that H_2_O_2_ exposure increases *LC3-II* and *P53* expression in granulosa cells while concurrently reducing *LC3-I* and *BCL-2* levels, resulting in heightened autophagy and apoptotic processes [10]. Notably, oxidative stress elevates the LC3-II/LC3-I ratio and Beclin-1 levels while diminishing P62 expression in goose granulosa cells [11]. Nevertheless, the optimal H_2_O_2_ concentration and exposure duration show significant variation depending on cell type and culture parameters [12], underscoring the necessity of establishing reliable oxidative damage models for antioxidant research.

Melatonin (MT) regulates circadian rhythms and ovarian functions, including follicular development, oocyte maturation, and oxidative damage reduction in granulosa cells [13,14]. It lowers ROS levels and boosts antioxidant enzymes like superoxide dismutase (SOD), catalase (CAT), and glutathione (GSH) [15]. MT also maintains mitochondrial function through its antioxidant, anti-apoptotic, and free radical-scavenging properties [16,17]. Xu’s research showed MT mitigates mitochondrial membrane potential decline in oxidative-damaged bovine granulosa cells [18].

Although previous poultry studies have highlighted MT’s potential in combating oxidative damage, its role in protecting hen follicular GCs remains unclear. This study cultured chicken follicular GCs in vitro, inducing oxidative damage with H_2_O_2_ to establish a model, and then identified the optimal exogenous MT concentration. It explores how MT alleviates GCs damage under oxidative damage by regulating mitochondrial autophagy. The findings provide insights into MT’s effects on poultry ovarian function.

## 2. Materials and Methods

### 2.1. Ethics Statement

All experimental protocols involving poultry subjects in this investigation received approval from the Institutional Animal Care and Use Committee (IACUC) at Jilin Agricultural University, Changchun, China [Approval No. GR (J) 19-89]. Research activities were performed in compliance with the ARRIVE guidelines [19]. Prior to organ harvesting, avian specimens underwent euthanasia via decapitation as mandated by IACUC standards for laboratory animals. This procedure was implemented in strict accordance with Chinese legislative requirements governing experimental animal welfare, specifically adhering to the 2017 revised edition of the Regulations for the Administration of Experimental Animal Affairs promulgated by the State Council of the People’s Republic of China.

### 2.2. Cell Culture

Following euthanasia, the abdominal feathers of the hens were plucked and the exposed area was thoroughly disinfected. The follicles were extracted and placed in pre-cooled sterile PBS (Biosharp, Anhui, Hefei, China) dishes. The follicular outer membrane, connective tissues, and vascular structures underwent meticulous dissection to separate them from the yolk mass. Forceps were utilized to stabilize the outer follicle membrane during GCs layer extraction. After thorough PBS washing, the dissected tissue was transferred to a sterile beaker for precise mincing. Minced specimens were subsequently loaded into 15 mL centrifuge tubes and subjected to centrifugation for supernatant removal. Enzymatic digestion was initiated by supplementing 7 mL collagenase type II (Biosharp, Anhui, Hefei, China), with the reaction proceeding for 15 min. Termination of digestion involved equal-volume complete medium addition followed by 15 min of centrifugation for cellular pellet collection. Post-filtration processing included cell resuspension in complete medium and subsequent plating into 6-well culture plates. Cellular cultures were maintained at 37 °C under 5% CO_2_ atmosphere for 12 h [20].

### 2.3. Oxidative Damage Model Cell Treatment

GCs obtained through isolation were plated into 96-well culture dishes and cultured under standard conditions (37 °C and 5% CO_2_). Following a 12 h attachment period, a systematic evaluation of H_2_O_2_ concentration gradients through experimental assays was implemented. For the control group, 0 μmol/L H_2_O_2_ was added; for the experimental groups, 5, 10, 20, 40, 80, 160, or 320 μmol/L H_2_O_2_ (diluted with complete medium) was added to treat the cells. The appropriate concentration range was determined by assessing cell viability and further screening was conducted to identify the optimal treatment concentration and duration within this range.

### 2.4. Cell Viability Assay

GCs were initially plated in 96-well culture dishes and maintained under standard culture conditions (37 °C, 5% CO_2_). Following a 12 h adhesion period, experimental groups received hydrogen peroxide treatments at predetermined concentrations (detailed in Methodology). Twenty microliters of CCK-8 reagent (APExBIO, Houston, TX, USA) were added per well, followed by light-protected incubation at physiological temperature for 60 min. Post-bubble elimination, cellular metabolic activity was quantified through 450 nm absorbance measurements using a microplate reader (Bio-Rad, Hercules, CA, USA), enabling the systematic evaluation of oxidative stress parameters on viability.

### 2.5. ELISA Assay

Quantitative analysis of the CAT, GSH, and SOD concentrations in GCs was conducted using commercial competitive ELISA kits (Lengton Bioscience Co., Shanghai, China). Post-treatment cellular samples were processed into PBS-diluted suspensions with a standardized density of 1 × 10^6^ cells/mL. Intracellular constituents were liberated through three consecutive freeze–thaw cycles. Following 15 min of centrifugation at 12,000× *g*, the resultant supernatant was harvested for analysis. Manufacturer-specified protocols were rigorously followed during assay execution. Optical density measurements at the 450 nm wavelength were acquired via microplate spectrophotometry, with subsequent data processing employing ELISAcale V-0.1 software’s four-parameter logistic curve fitting algorithm.

### 2.6. RNA Isolation and Quantitative Reverse Transcription PCR (qRT-PCR) Analysis

Follicular GCs RNA extraction was performed employing Trizol reagent (Biosharp, Anhui, Hefei, China). Subsequently, purified RNA underwent reverse transcription using the ToloScript All-in-one RT EasyMix for qPCR kit (Tolobio, Shanghai, China) in 20 µL reaction systems. cDNA amplification was conducted in 20 µL reactions containing SYBR Green qPCR Mix (Biosharp, Anhui, Hefei, China). Target gene mRNA expression quantification was achieved through quantitative reverse transcription PCR analysis. Gene-specific primers were computationally designed via Primer Premier 5 software and commercially synthesized by Sangon Biotech (Shanghai, China). β-actin served as the endogenous control, with the corresponding primer sequences detailed in Table 1. Thermal cycling parameters comprised an initial denaturation (95 °C, 10 min), followed by 40 cycles of denaturation (95 °C, 15 s) and primer annealing/extension (60 °C, 60 s). Co-amplification of β-actin and target genes occurred within identical reaction chambers for individual samples, with each analysis implemented in triplicate technical replicates across two independent experimental runs. Normalization of threshold cycle (Ct) values against the reference gene enabled relative quantification through 2 − (ΔCt_sample − ΔCt_control) calculation [14].

### 2.7. Western Blotting

Post-treatment cellular material was harvested from culture vessels with subsequent removal of growth medium. Cellular washing procedures involved 1 mL PBS application. Lysis buffer (200 μL RIPA (Biosharp, Anhui, Hefei, China) supplemented with protease/phosphatase inhibitors) was introduced for protein extraction. Following detachment, cellular suspensions were collected into 1.5 mL tubes via scraping. Lysates underwent centrifugation (12,000 rpm, 10 min, 4 °C) to obtain clarified supernatants. Protein concentrations were determined through bicinchoninic acid (BCA) assay. Aliquots were mixed with 5× loading buffer, followed by thermal denaturation (95 °C, 5 min) to achieve structural modification. Electrophoretic resolution of denatured proteins occurred through SDS-PAGE gels. Post-separation transfer onto PVDF membranes was implemented. Membranes received blocking treatment through immersion in TBST containing 5% skim milk (2 h, ambient temperature). Primary antibody incubation (anti-P62, LC3-I, LC3-II, and GAPDH) (Proteintech, Hubei, Wuhan, China) proceeded overnight. Following TBST washes, a secondary antibody exposure was conducted (2 h, room temperature). Chemiluminescent substrates were subsequently applied for signal development, with visualization accomplished using a multifunctional gel documentation platform. Band intensities were analyzed using AlphaEaseFC 4.0 software and quantitative analysis was performed by plotting histograms.

### 2.8. Autophagy Detection

Post-treatment cellular harvesting was achieved through trypsinization followed by centrifugation (1200 rpm, 3 min) with subsequent supernatant removal. The pelleted cells underwent PBS resuspension and washing procedures prior to repetition of centrifugation parameters. AO (Bestbio, Shanghai, China) staining reagent preparation strictly followed manufacturer-provided protocols. Cellular suspensions (95 μL) were combined with 5 μL AO staining solution and dark-incubated at 4 °C for 15 min. Post-incubation processing included dual staining buffer washes and centrifugation (1200 rpm, 3 min, 4 °C), culminating in final resuspension within staining buffer.

### 2.9. Mitochondrial Membrane Potential Detection

Post-treatment cellular collection involved trypsin digestion followed by centrifugation (1200 rpm, 3 min) with subsequent supernatant removal. The harvested cellular precipitate underwent PBS resuspension and washing procedures before repeating centrifugation under equivalent parameters. JC-1 (Bestbio, Shanghai, China) dye working solution preparation required sequential addition of 50 μL 200× stock solution to 8 mL deionized water with thorough mixing through vortex agitation, followed by supplementation with 2 mL 5× JC-1 staining buffer. Cellular suspensions (0.5 mL working solution) were incubated at 37 °C for 20 min. Post-incubation processing included dual washes with 1× JC-1 buffer and centrifugation (1200 rpm, 3 min, 4 °C), concluding with final resuspension in 1× JC-1 buffer.

### 2.10. Data Analysis

Experimental data presentation utilized mean values with standard error (SE). Statistical evaluations were performed through SPSS 23.0 (IBM Corporation) utilizing single-factor ANOVA methodologies to determine group variations after verifying variance homogeneity. For post hoc testing, the Bonferroni test was selected to ensure the reliability of the data. All experimental designs incorporated a minimum of three independent biological replicates. Probability thresholds for statistical relevance were established at *p* < 0.05 (significant) and *p* < 0.01 (marked significance).

## 3. Results

### 3.1. H_2_O_2_-Induced Granulosa Cell Oxidative Damage Model

**Figure 1:**
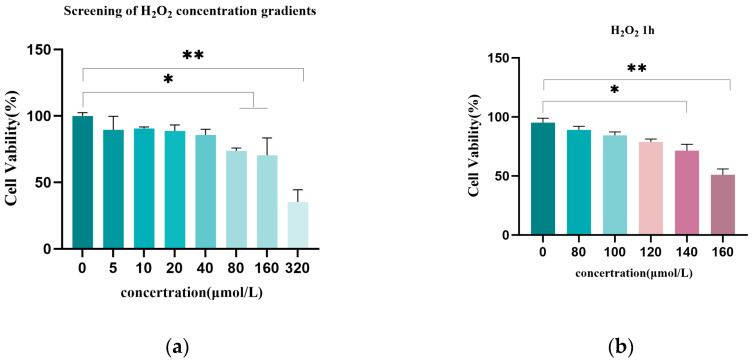

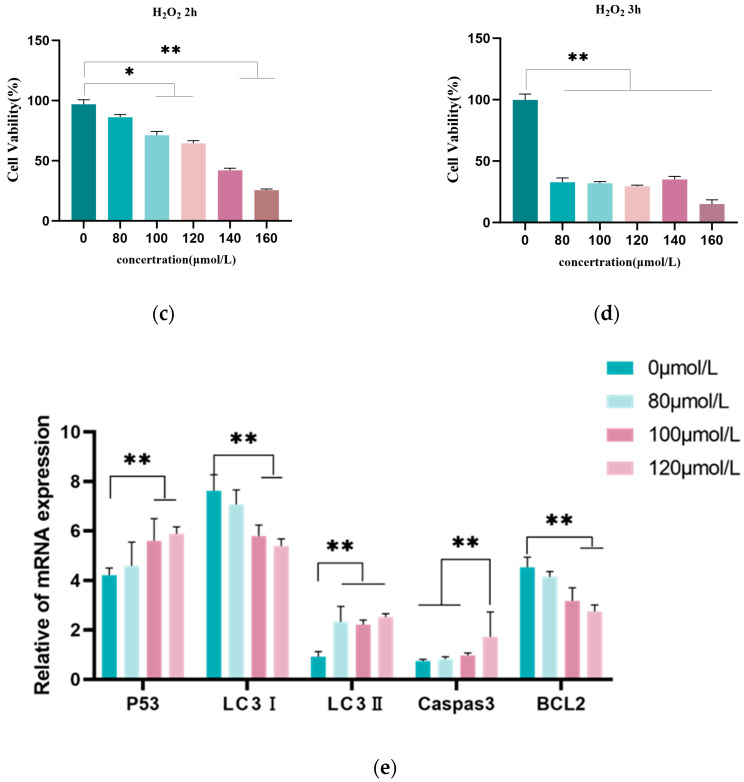
Establishment of the H_2_O_2_-induced GCs oxidative damage model. Screening of the optimal H_2_O_2_ treatment concentration and time for inducing oxidative damage in GCs. GCs were exposed to varying concentrations of H_2_O_2_. (**a**–**d**) CCK-8 assay outcomes, where cellular viability was quantified through 450 nm absorbance measurements using microplate spectrophotometry. Viability calculations followed standardized formulae, (**e**) quantitative PCR analysis of autophagy regulators (*P53*, *LC3-I*, and *LC3-II*) and apoptosis modulators (*Caspase-3* and *BCL-2*) across varying H_2_O_2_ concentrations. The untreated group (0 μmol/L H_2_O_2_) served as a blank control, with *β-actin* functioning as an endogenous reference. Triplicate biological replicates were conducted, with results presented as mean ± S.E (n = 3). Statistical annotations: * *p* < 0.05, ** *p* < 0.01 versus control.

To establish an oxidative damage model, this study used the CCK-8 assay to analyze follicular GCs viability and qRT-PCR to measure autophagy- and apoptosis-related gene expression.(Figure 1a–e) showed the results that 80–160 μmol/L H_2_O_2_ significantly reduced cell viability (*p* < 0.05), with 320 μmol/L causing a severe decline (*p* < 0.01, survival < 50%). After 2 h, 100–120 μmol/L H_2_O_2_ significantly decreased viability (*p* < 0.05), while 140–160 μmol/L caused a more pronounced reduction (*p* < 0.01, survival < 50%). qRT-PCR revealed that 100 μmol/L H_2_O_2_ substantially upregulated *p53* and *LC3-II* (*p* < 0.05) and decreased *LC3-Ⅰ* (*p* < 0.05), with no significant changes in *Caspase-3* or *BCL-2* (*p* > 0.05). Thus, 100 μmol/L H_2_O_2_ for 2 h was selected as the suitable condition for the oxidative damage model.

### 3.2. Screening of the Optimal Melatonin Concentration for Relieving Oxidative Damage in Model Cells

**Figure 2:**
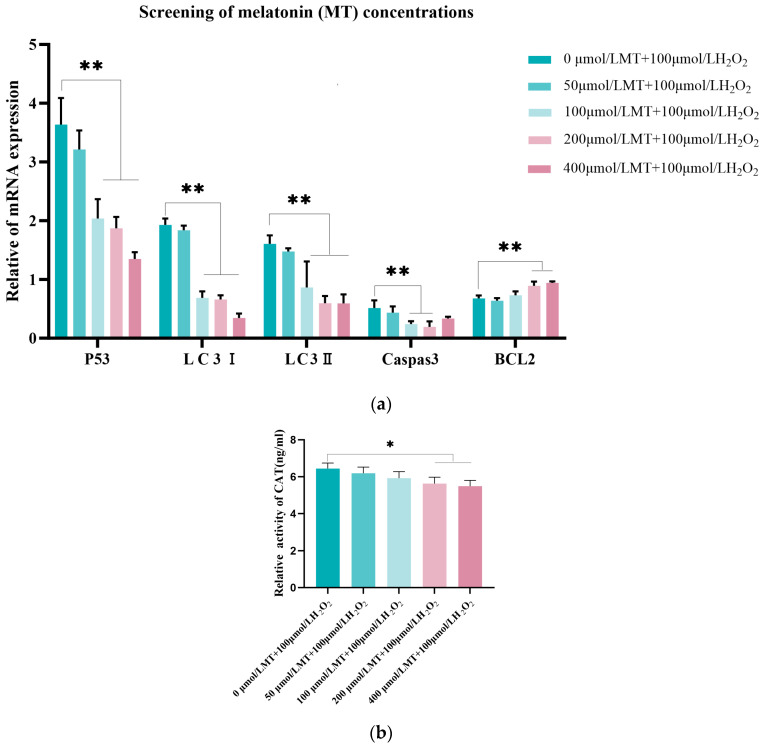
Optimization of treatment concentration for H_2_O_2_-stimulated GCs. (**a**) Quantitative analysis using fluorescent methodologies evaluated expression variations in autophagy regulators (*P53*, *LC3-I*, and *LC3-II*), anti-apoptotic mediator (*BCL-2*), and apoptosis-associated protein (*Caspase-3*), normalized against *β-actin* as the endogenous control. (**b**) CAT enzymatic activity quantification via ELISA. Triplicate experimental replicates were conducted, with results presented as mean ± standard error (S.E; n = 3). Statistical annotations: * denotes statistically significant differences versus control (*p* < 0.05); ** indicates highly significant variations (*p* < 0.01).

To determine the optimal MT concentration for alleviating oxidative damage in the model cells, the 100 μmol/L H_2_O_2_-treated cells were used as the control group. qRT-PCR was performed to analyze autophagy-related factors (*P53*, *LC3-Ⅰ*, and *LC3-Ⅱ*) expression and an ELISA kit was used to measure the changes in CAT levels in the cells. As depicted in (Figure 2a) quantitative analysis revealed comparable expression levels of autophagy-associated markers (*P53*, *LC3-I*, and *LC3-II*) between the 50 μmol/L MT-treated group and the model group (*p* > 0.05). Significant suppression of *Caspase-3* expression was observed in both 100 μmol/L and 200 μmol/L MT administration groups (*p* < 0.01), whereas upregulation of the anti-apoptotic mediator *BCL-2* reached statistical significance in the 200 μmol/L and 400 μmol/L treatment cohorts (*p* < 0.01). Downregulation trends for autophagy regulators were evident in MT concentrations of 100 μmol/L, 200 μmol/L, and 400 μmol/L. (Figure 2b) demonstrates ELISA quantification results showing substantially diminished intracellular CAT content in 200 μmol/L and 400 μmol/L MT groups relative to model controls (*p* < 0.05). Based on these findings, the 200 μmol/L MT concentration was ultimately chosen as the optimal therapeutic dosage for subsequent experimental protocols.

### 3.3. Melatonin Alleviates the Initiation of Autophagy in Granulosa Cells

**Figure 3:**
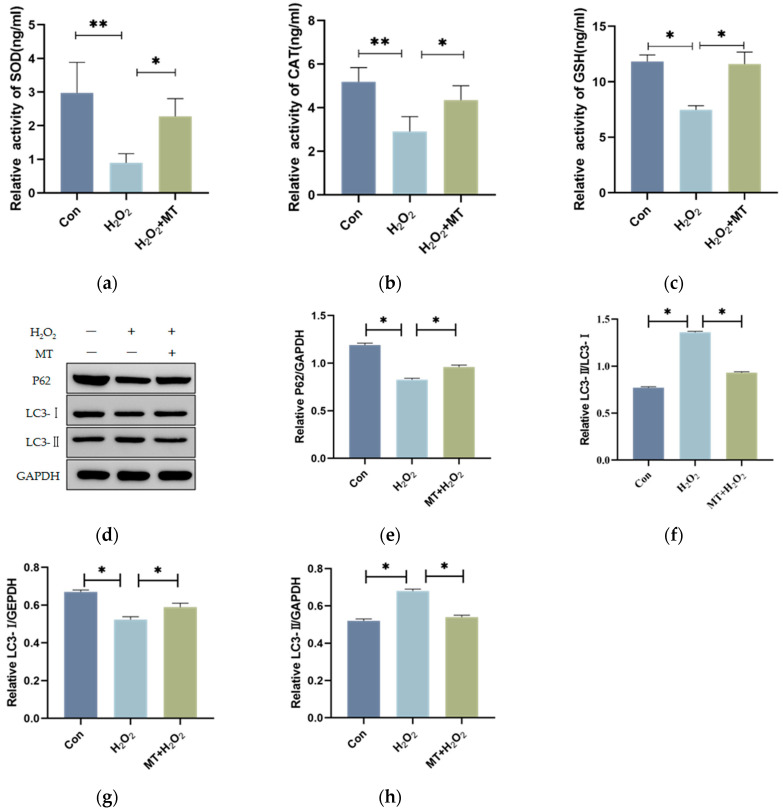
MT inhibits autophagy in GCs under oxidative damage. GCs were cultured for 12 h, followed by treatment with 100 μmol/L H_2_O_2_ for 2 h as the oxidative damage model group. The treatment group received 200 μmol/L MT co-treated with H_2_O_2_ for 2 h, representing the optimal MT concentration. (**a**–**c**) ELISA was used to measure oxidative damage markers such as CAT, SOD, and GSH, with the blank group serving as the control. The experiment was repeated 3 times. (**d**–**h**) Immunoblot analyses identified autophagy-associated proteins (P62, LC3-I, and LC3-II) in cellular lysates, normalized against GAPDH expression. Quantitative data presentation utilized mean ± SE values (n = 3). Statistical significance: ** *p* < 0.01 (highly significant vs. control); * *p* < 0.05 (significant vs. control).

To further investigate the potential regulatory role of MT, we examined whether it could alleviate autophagy in follicular GCs under oxidative damage. Cells were subjected to repeated freeze–thaw cycles to induce cell lysis and oxidative damage markers in GCs were detected using an ELISA kit. Experimental findings presented in Figure 3a–c demonstrate that H_2_O_2_ exposure induced a significant reduction in SOD and CAT concentrations, while GSH levels exhibited a substantial decrease compared to the control specimens. Conversely, combined administration of H_2_O_2_ and MT resulted in enhanced SOD, CAT, and GSH activities. Western blot analyses (Figure 3d–h) revealed diminished LC3-II protein abundance alongside elevated P62 expression in H_2_O_2_-treated cohorts, with concurrent inhibition of LC3-I to LC3-II conversion. These H_2_O_2_-induced molecular alterations were counteracted by MT co-treatment. Collectively, the data support MT’s capacity to mitigate autophagic initiation mechanisms in GCs.

### 3.4. Melatonin Alleviates Mitochondrial Autophagy-Induced Oxidative Damage in Granulosa Cells (GCs)

**Figure 4:**
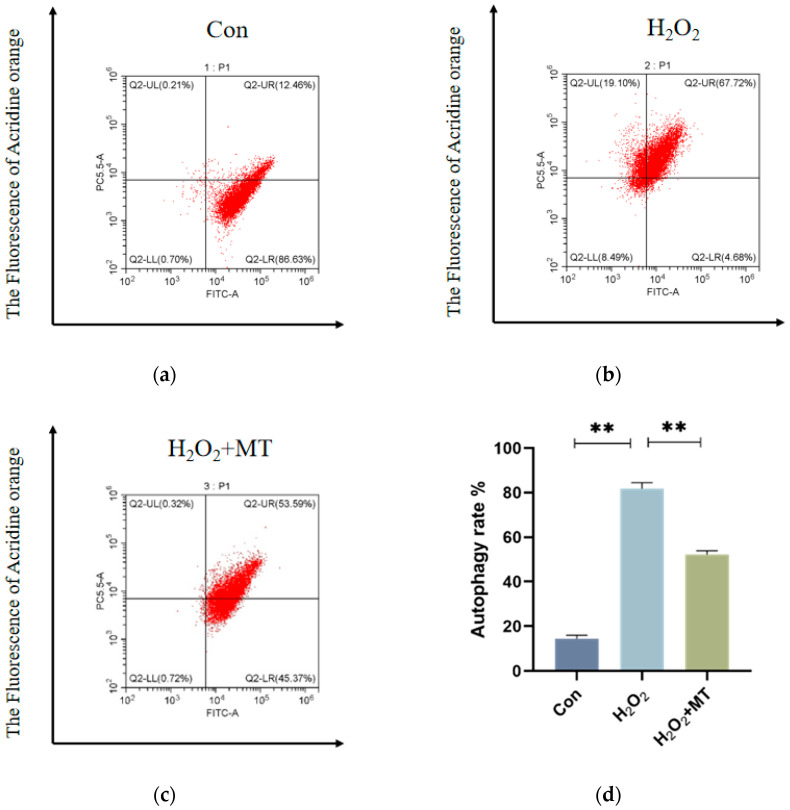

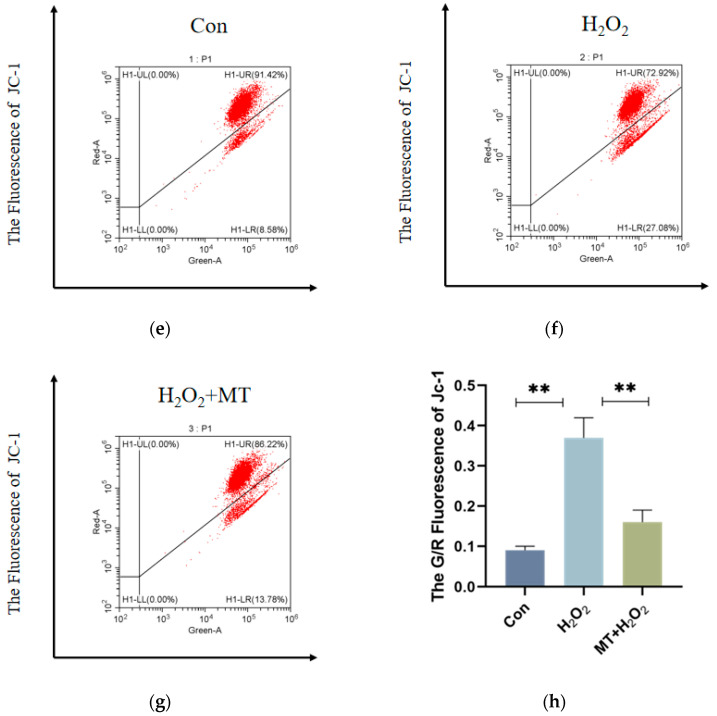
MT alleviates H_2_O_2_-induced mitochondrial damage in GCs. Acridine orange staining and flow cytometry were used to detect acidic autophagic vacuoles within the cells. (**a**–**c**) The flow cytometry plot shows the *X*-axis representing the FITC channel signal (green fluorescence) and the PC5.5 channel signal (red fluorescence). The density of the red scatter points reflects the change in the count of autophagic vacuoles in the cells. After H_2_O_2_ treatment, the fluorescence signal significantly increased, while MT treatment reduced the signal intensity. (**d**) JC-1 staining was used to assess the mitochondrial membrane potential (ΔΨm) by flow cytometry. The *X*-axis represents the FITC channel (green fluorescence) and the *Y*-axis represents the PE channel (red fluorescence). (**e**–**g**) The autophagy rate is represented by the sum of the values in the Q2-UL and Q2-UR quadrants. (**h**) The JC-1 red-to-green fluorescence ratio (G/R) of different treatment groups is shown to reflect changes in mitochondrial membrane potential. The experiment was conducted in triplicate, with data shown as mean ± SEM. ** *p* < 0.01.

To explore whether MT can reduce mitophagy in GCs exposed to oxidative damage. Experimental data presented in (Figure 4a–c) demonstrate that H_2_O_2_ exposure induced a significant elevation (*p* < 0.01) of PC5.5 channel fluorescence intensity compared to untreated controls, whereas MTadministration substantially attenuated this signal enhancement. (Figure 4h) reveals a pronounced decline in mitochondrial membrane potential coupled with a marked increase in G/R ratio (*p* < 0.01) in H_2_O_2_-exposed specimens relative to baseline measurements. Notably, combined H_2_O_2_/MT treatment effectively mitigated mitochondrial membrane potential deterioration, concurrently reducing the G/R ratio to levels significantly below those observed in the H_2_O_2_-only group (*p* < 0.01). These observations collectively indicate MT’s therapeutic potential in ameliorating H_2_O_2_-induced mitochondrial functional impairments.

## 4. Discussion

Oxidative damage plays a role in physiological processes and disease onset [21]. H_2_O_2_ easily penetrates cell membranes, causing oxidative damage, and is widely used in models [22,23]. Excessive ROS presence during follicle development reduces follicle numbers and reproductive performance in poultry [24]. H_2_O_2_ concentrations and exposure times vary by cell type and culture conditions. Zheng et al. established an oxidative damage model in goose GCs using 250 μmol/L H_2_O_2_ for 6 h [7]. ROS accumulation induced apoptosis, blocked autophagic flux, and led to follicular atresia. This study aimed to trigger autophagy while maintaining cell viability. While low-dose H_2_O_2_ (50 μmol/L) exerted minimal effects, higher concentrations (150–250 μmol/L) triggered a marked downregulation of *BCL-2*, a significant upregulation of *Caspase-3*, and a sharp decline in cell viability below 50%, causing irreversible cell death. Based on viability and autophagy factor expression, 100 μmol/L H_2_O_2_ for 2 h was selected as the optimal condition for oxidative damage modeling in chicken GCs.

Changes in the antioxidant system reflect cellular damage [5,20,25]. Mitochondrial H_2_O_2_ removal relies on the GSH peroxidase system (Gpx1 and mGpx4) and peroxiredoxins (Prx3 and Prx5) [26]. Bai et al. [27] found MT inhibits oxidative damage and enhances antioxidant enzyme activity (SOD, CAT, and GSH-Px), consistent with this study. H_2_O_2_ treatment significantly reduced SOD, CAT activity, and GSH levels in GCs, indicating oxidative damage. However, these parameters were restored by MT co-treatment, suggesting that oxidative damage induced by H_2_O_2_ is counteracted by MT through the enhancement of cellular antioxidant capacity.

Poultry egg production depends on follicle numbers, with follicular atresia primarily caused by GCs apoptosis [28]. Autophagy supports follicular development but, when excessive, induces cell death and atresia. Increased GC autophagy occurs during follicular atresia in mice and geese [29,30,31]. As a key metabolic process, autophagy prevents damage by degrading waste but can also cause cell death if excessive [32]. The results demonstrated that H_2_O_2_ treatment markedly increased the number of autophagic vacuoles in GCs, whereas MT supplementation significantly this effect, indicating that MT partially suppressed H_2_O_2_-induced autophagy.

Additionally, P62, a key autophagy receptor, recognizes phosphorylated polyubiquitin chains on mitochondrial proteins and binds LC3-II to initiate autophagosome formation [33,34]. P62 accumulates when autophagic flux is inhibited and decreases when activated. Wang et al. [35] found bovine GC autophagy marked by increased Beclin-1 and LC3-II/LC3-I levels and decreased P62. This result demonstrated that P62was reduced by H_2_O_2_, while the LC3-II/LC3-I ratio was increased, indicating that autophagy was induced. These effects were reversed by MT, suggesting that the excessive autophagy induced by H_2_O_2_ was mitigated.

Mitochondria, the main ROS source, play a key role in oxidative damage, autophagy, and apoptosis [36,37]. Mitochondrial autophagy selectively removes damaged mitochondria [38] and its extent is linked to the reproductive decline in laying hens [39,40]. Excessive ROS damages the mitochondrial membrane, reducing membrane potential (, causing depolarization, increasing permeability, and disrupting redox balance [41,42]. Membrane potential changes signal early mitochondrial dysfunction [43]. JC-1 staining and flow cytometry showed that an increase in green fluorescence in GCs was induced by H_2_O_2_, indicating mitochondrial damage, while depolarization was alleviated by MT, protecting GCs through the inhibition of mitochondrial autophagy. In agreement with findings from mammalian studies, this MT reduces oxidative stress in ovarian mitochondria through three primary mechanisms: decreasing mROS generation, inhibiting programmed cell death pathways, and preserving mitochondrial membrane integrity [44]. A foundation for exploring the role of MT in mitochondrial regulation is provided by this study.

The main limitation of this study is the lack of a systematic measurement of baseline endogenous MT levels in GCs or dynamic changes in hormone profiles. The absence of these data hindered a deeper exploration of MT’s natural regulatory mechanisms and their association with its antioxidative protective effects. However, the findings suggest that MT may indirectly enhance poultry egg production by maintaining follicular health, thereby providing a theoretical basis for developing MTbased feeding strategies to optimize reproductive performance.

## 5. Conclusions

It was demonstrated in this study that oxidative damage induced by hydrogen peroxide in chicken follicular GCs is alleviated by MT. This effect is achieved through the mitigation of intracellular antioxidant enzyme activities (SOD, CAT, and GSH), a reduction in the LC3-II/LC3-I ratio, an increase in P62 protein expression, a decrease in the formation of intracellular autophagic vesicles, and an enhancement of mitochondrial membrane potential.

## Figures and Tables

**Table 1:** Primers for PCR analysis.

Gene	Forward	Reverse
*BCL2*	GGACGCTTGGCTATCCCTAC	CTATGATGCGATGGCACGAC
*Caspase3*	GCTGAAGGCTCCTGGTTTAT	TTCTGCCACTCTGCGATTTA
*LC3-I*	TTACACCCATATCAGATTCTTG	ATTCCAACCTGTCCCTCA
*LC3-II*	AGTGAAGTGTAGCAGGATGA	AAGCCTTGTGAACGAGAT
*P53*	GCAAGATCGAGGAGGAGAACT	ATCTCATTGTCGGGGTTCAG
*β-actin*	GCCAACAGAGAGAAGATGACACAG	CATCACCAGAGTCCATCACAATACC

## Data Availability

The original contributions presented in this study are included in this article. Further inquiries can be directed to the corresponding authors.

## Abbreviations

H_2_O_2_	hydrogen peroxide
LC3-I	Microtubule-associated Protein 1A/1B-light Chain 3-I
LC3-II	Microtubule-associated Protein 1A/1B-light Chain 3-II
CAT	catalase
GSH	glutathione
SOD	superoxide dismutase
MT	melatonin
ROS	reactive oxygen species
P62	Sequestosome 1
GCs	granulosa cells
Ct	cycle threshold
